# Diagnostic accuracy of the HUAWEI WATCH D for measuring spinal anesthesia-induced hypotension during elective cesarean section: a diagnostic test

**DOI:** 10.3389/fmed.2025.1686637

**Published:** 2026-01-14

**Authors:** Wei Li, Yan Huan, Xianjin Zhou, Shuangqiong Zhou, Haibing Li, Na Wu, Fuyi Shen

**Affiliations:** 1 Shanghai Key Laboratory of Maternal-Fetal Medicine, Department of Anesthesiology, Shanghai Institute of Maternal-Fetal Medicine and Gynecologic Oncology, Shanghai First Maternity and Infant Hospital, School of Medicine, Tongji University, Shanghai, China; 2 Shanghai Key Laboratory of Maternal-Fetal Medicine, Department of Nursing, Shanghai Institute of Maternal-Fetal Medicine, and Gynecologic Oncology, Shanghai First Maternity and Infant Hospital, School of Medicine, Tongji University, Shanghai, China

**Keywords:** cesarean section, combined spinal epidural anesthesia, diagnostic test, HUAWEI WATCH D, hypotension

## Abstract

**Background:**

The newly developed HUAWEI WATCH D (HW) is a wearable smartwatch, primarily designed to measure non-invasive blood pressure (NIBP) at the wrist. Two recent studies have demonstrated that the accuracy of HW is the same as that of mercury sphygmomanometer for measuring BP in adults. This study aimed to validate the accuracy of the HW in hypotension monitoring.

**Methods:**

A total of 118 healthy parturients with singleton pregnancies undergoing elective cesarean section were included. BP was measured three times preoperatively on the left wrist using HW and on the right upper limb using an Omron BP monitor, with the respective mean values recorded as the baseline values in the supine position. After spinal anesthesia, bilateral BP was measured simultaneously by HW and a NIBP monitor at 1-min intervals, 10 times. The primary outcome was the Chi-squared test results comparing hypotension detected between HW and NIBP, as well as the area under the curve (AUC) for receiver operating characteristic (ROC), the Youden index, and the cut-off point of HW for measuring hypotension.

**Results:**

Overall, 1180 pairs of systolic blood pressure (SBP) were measured. Using an SBP of <80% baseline values of NIBP as the gold standard for hypotension, Youden Index = 0.62, the hypotension threshold for HW is set to 104 mmHg, with sensitivity of 79.2%, specificity of 82.0%, false positive rate of 18.0%, and false negative rate of 20.8%, and accuracy of 81.3%. The Chi-squared test yielded a Pearson γ^2^ = 350.4, Kappa = 0.53, *P* < 0.001 (two-tailed). The AUC of ROC was 0.87 (95% confidence interval: 0.85–0.90), *P* < 0.001 (two-tailed).

**Conclusion:**

The HUAWEI WATCH D demonstrates moderate diagnostic accuracy for detecting severe hypotension (SBP < 80% baseline) during caesarian section under spinal anesthesia, with a sensitivity of 79.2%, a specificity of 82.0%, and an AUC of 0.87.

## 1 Introduction

The newly developed HUAWEI WATCH D (HW) (Huawei Terminal Co., Ltd., Shenzhen, China) is a smartwatch that can monitor various physical indicators, including heart rate, electrocardiogram, blood oxygen saturation, and sleep duration. Its primary function is to measure non-invasive blood pressure (NIBP) at the wrist. HW is a smartwatch equipped with a built-in airbag that measures BP using the oscillometric method. The measurement range of the HW is 60–230 mmHg for SBP and 40–160 mmHg for DBP. This range covers the normal BP range of pregnant women. So, we believe it can be used for BP measurement research in pregnant women. HW determines SBP and DBP by an algorithm analyzing the pulse wave detected during inflation (1). It has obtained the Class II medical device registration inspection and met international standards (2, 3).

Owing to its compact, portable, and smart design with smartphone connectivity, we believe it can be flexibly utilized in scenarios outside operating rooms or hospital ward where traditional NIBP is unavailable.

For non-pregnant adult populations, two studies have demonstrated that the HW provides accurate BP measurements (1, 4). As a new wearable device, its accuracy in measuring hypotension should also undergo extensive validation.

Given that some studies report the incidence of hypotension as high as 90% following combined spinal-epidural anesthesia (CSEA) for elective cesarean sections (5, 6). If the monitoring device delays displaying hypotension readings, it may lead to the occurrence of supine hypotension syndrome symptoms in parturients. Parturients may experience nausea, vomiting, tachycardia, reduced placental blood supply, fetal hypoxia and acidosis (7). We aimed to evaluate the accuracy of HW in measuring hypotension specifically in parturients undergoing elective cesarean delivery under neuraxial anesthesia.

## 2 Materials and methods

### 2.1 Study participants

#### 2.1.1 Ethics statement

This study has been certified by the Ethics Committee with registration number KS23267 (registered institution: Shanghai First Maternity and Infant Hospital; Ethics Chairman: Luo Ye) and registered with the Clinical Trial Registration Center (Chi CTR 2400079421). The research duration was from 20 January 2024 (20/01/2024) to 7 August 2024 (07/08/2024). This trial was prospectively registered prior to patient enrollment.

#### 2.1.2 Inclusion criteria

(1) American Society of Anesthesiologists physical status I – II; (2) age, 18 – 45 y; (3) full-term pregnant women scheduled for elective cesarean delivery; (4) singleton pregnancy (5) anesthetized with CSEA; and (6) signed informed consent.

#### 2.1.3 Exclusion criteria

(1) Women declining participation; (2) inter-arm SBP difference > 10 mmHg per monitor measurement; (3) conversion to general anesthesia; (4) allergic reactions; (5) gestational hypertension; (6) constitutional hypotension; (7) contraindications to neuraxial anesthesia; (8) total spinal anesthesia, exceptionally extensive spinal block, or inadequate blockade; and (9) emotional lability upon entering the operating room.

According to the 2018 Advancement of Medical Instrumentation (AAMI)/European Society of Hypertension/ International Organization for Standardization (ISO) consensus guideline requiring 85 cases (3), our final sample size was set at 118 participants. The AAMI/ESH/ISO guideline was the primary basis for sample size.

### 2.2 CSEA

A standardized protocol for CSEA was followed. With the parturient in the right lateral position, an 18-gauge Tuohy needle was inserted into the epidural space at the L2–3 interspace. Using a needle-through-needle technique, a 25-gauge Whitacre needle was inserted intrathecally and 3 mL (15 mg) of 0.5% isobaric ropivacaine hydrochloride was injected. An epidural catheter was threaded 3–5 cm into the epidural space to facilitate augmentation of spinal anesthesia, if required. Parturients were positioned supine with their heads on a common pillow. Sensory block level was assessed as being to T6 or higher to cold stimulus 10 min after spinal injection (8). An epidural top-up was administered if the block failed to reach T6 within 10 min, or when the parturient reported pain. Conversion to general anesthesia was performed for a neuraxial block that was unsatisfactory after epidural supplementation.

### 2.3 Blood pressure measurement method

The parturient was wheeled to the operating room waiting area on a gurney, lying on supine position and resting on the trolley for 5 min, and the brachial artery BP of the left and right upper limbs was measured once using an Omron (Kyoto, Japan) BP monitor. If the difference in SBP between the upper limbs was ≤10 mmHg, the Omron BP monitor cuff was tied to the right upper limb, and a HW was worn on the left wrist. BP was measured every 1 min for three consecutive measurements, and the mean value of these three SBP readings was taken as the baseline values.

The parturient entered the operating room, received oxygen therapy, opened the vein, and received lactate Ringer’s solution. Measurements of the NIBP on the right upper limb and the HW on the left wrist were recorded. BP, heart rate (HR), and peripheral oxygen saturation (SPO_2_) were measured. The parturient was in the right lateral position and conducted CSEA in the L2–3 interval. The parturient was then positioned supine, with both upper limbs extended horizontally and placed on armrests. Bilateral BP was measured synchronously at an interval of 1 min, and the cold sensation of the block plane was gauged with an alcohol cotton ball 10 min later. Supine hypotensive syndrome (SHS) has a high incidence, and occurs 3–10 min after spinal anesthesia in supine position (9). The gold standard for post-anesthesia hypotension is SBP measured by NIBP that is <80% of the baseline value (10, 11). If the SBP was <80% of the baseline, phenylephrine (40–120 μg) or ephedrine (5–10 mg per instance) was injected intravenously to maintain the SBP within 80%–100% of the baseline (2, 5).

### 2.4 Statistical analysis

All manually recorded data was checked and entered into Excel (Microsoft, Redmond, WA, USA) text and SPSS 26.0 (IBM Corp., Armonk, NY, USA). Single sample *t*-test was used for quantitative data. Chi-square test was used for the accuracy of measuring hypotension with HW. Statistical significance was set at *P* < 0.05. Due to the shaking of that parturient’s arm, HW blood pressure measurement failed once, resulting in one missing value, which was replaced by the average of adjacent time points. This approach was deemed appropriate given the stability of the BP measurements at adjacent 1-min intervals in the perioperative period.

## 3 Results

**FIGURE 1:**
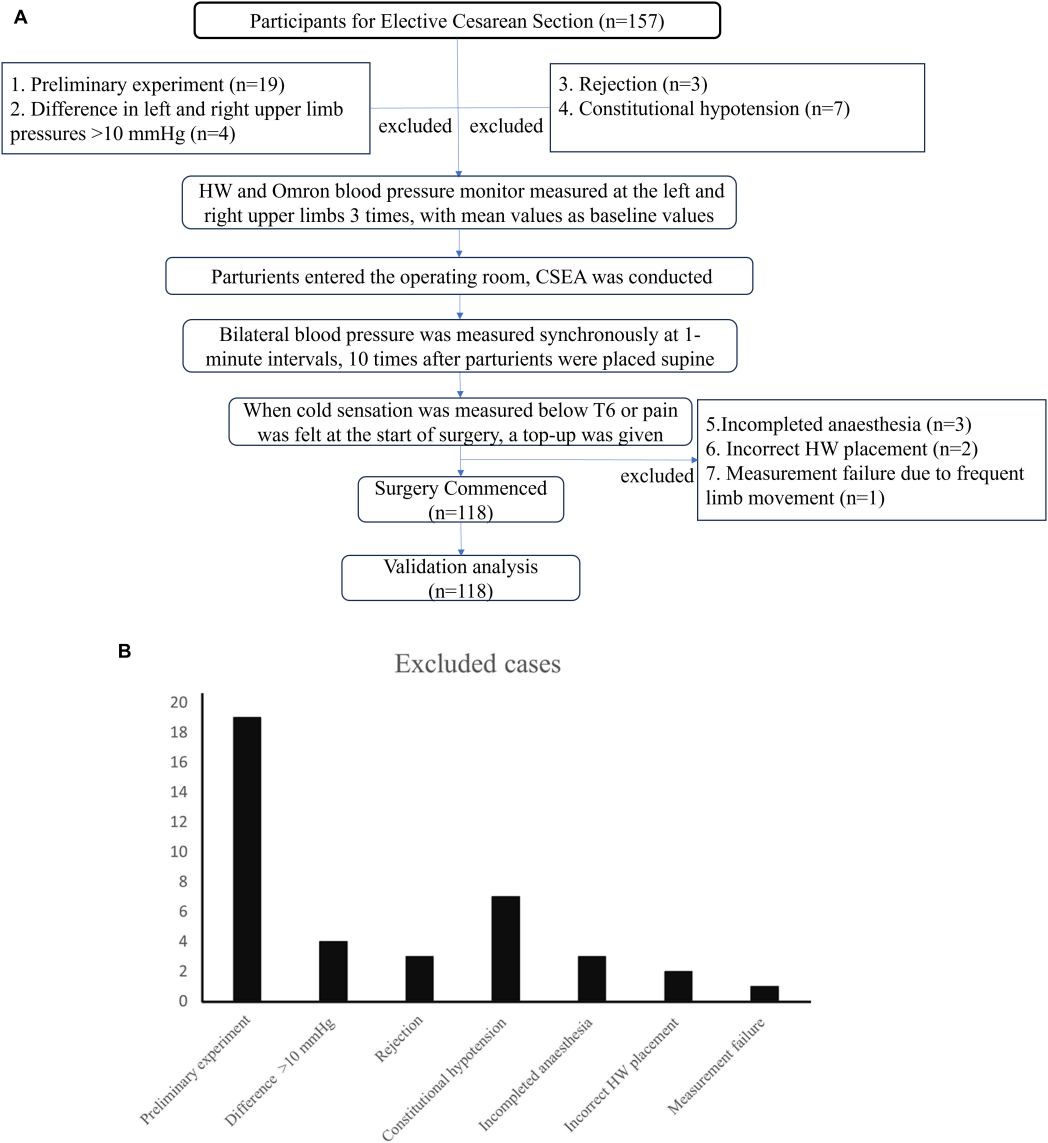
**(A)** Study flow diagram. **(B)** Legend of excluded cases.

This study included a total of 157 parturients who received spinal anesthesia for elective cesarean section in our hospital, of which 118 cases were obtained by removing those who: (1) were preliminary experiments (*n* = 19); (2) had a left and right upper limb SBP difference of >10 mmHg (*n* = 4); (3) were rejected (*n* = 3); (4) had constitutional hypotension (*n* = 7); (5) incomplete anesthesia (*n* = 3); (6) incorrect HW placement (*n* = 2); and (7) with measurement failure due to frequent limb movement (*n* = 1) (Figure 1). Written informed consent was obtained from all enrolled participants.

At all time-points, only the data from the final time-point failed to pass the normality test. For large-sample quantitative data (typically *n* > 30) that approximately follows a normal distribution, the *t*-test can be confidently applied–this is a statistically recommended approach.

### 3.1 Participant characteristics

Quantitative data meets the requirements of both homogeneity of variance and normality tests. The mean age of these participants was 32.0 ± 3.4 y, with a mean height of 161.2 ± 5.8 cm, a mean weight of 70.5 ± 12.9 kg, a mean body mass index (BMI) of 27.1 ± 4.8 kg/m^2^, and mean gestational age of 38.9 ± 1 weeks, the baseline SBP values at the left and right upper arms were 122.4 ± 8.1 and 123.7 ± 7.8 mmHg, respectively (r^2^ = 0.81, *P* < 0.001; Table 1).

**TABLE 1:** Demographics of parturients.

Items	M ± SD
Age (y)	32.0 ± 3.4
Height (cm)	161.2 ± 5.8
Weight (kg)	70.5 ± 12.9
BMI (kg/m^2^)	27.1 ± 4.8
Normal (*n* = 33)	23.2 ± 1.0
Overweight (*n* = 67)	27.1 ± 1.5
Obese (*n* = 18)	32.1 ± 2.1
Gestation weeks (W)	38.9 ± 1
SBP of left upper arm (mmHg)	122.4 ± 8.1
SBP of right upper arm (mmHg)	123.7 ± 7.8*

*r = 0.81, *P* < 0.001. SD, standard deviation; BMI, body mass index; SBP, systolic blood pressure.

**FIGURE 2:**
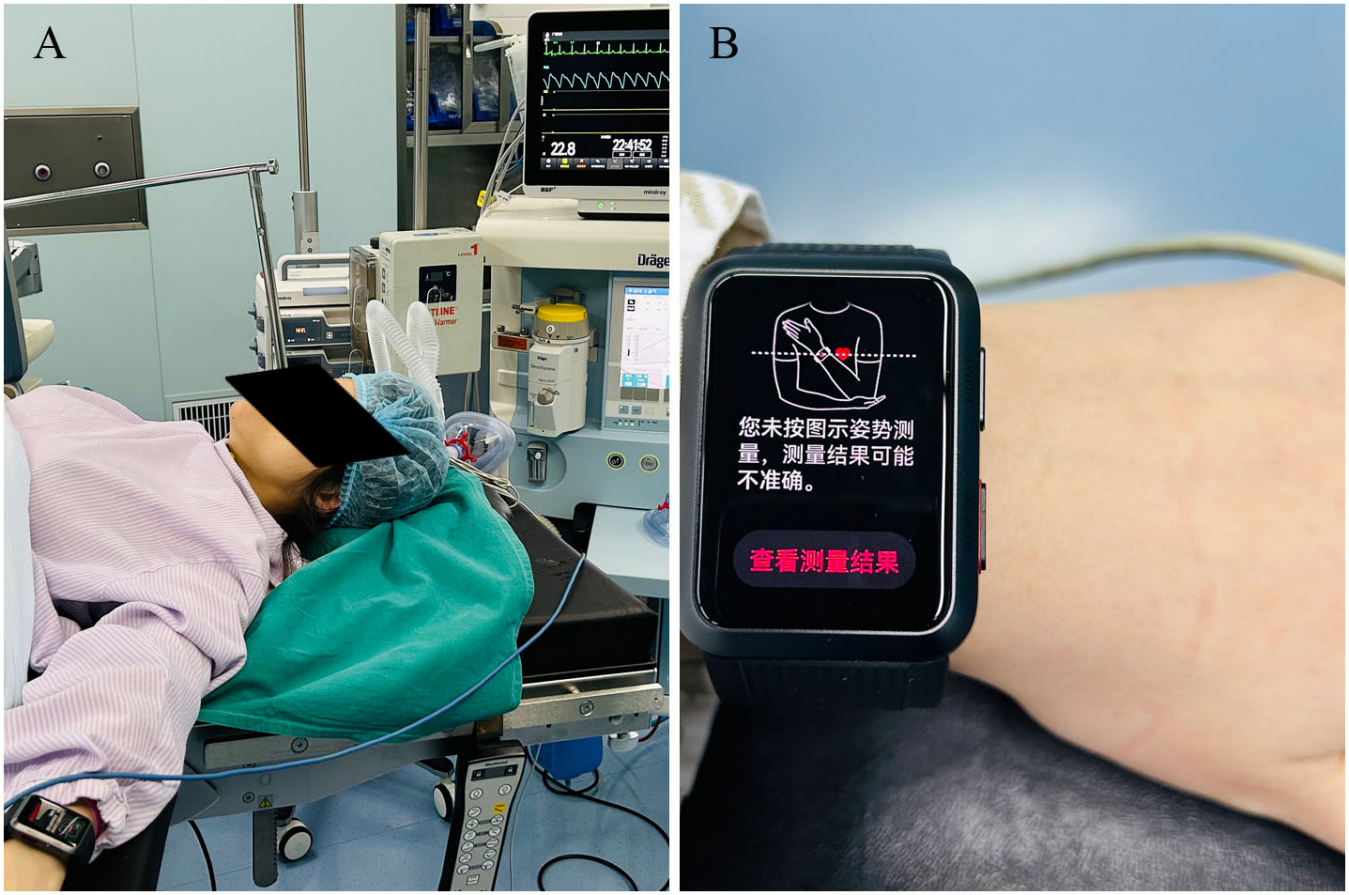
Supine positioning during BP measurement. **(A)** Intraoperative setup showing the patient in supine position with arms extended on armrests at body level during cesarean section. **(B)** HUAWEI WATCH D display showing position warning.

However, we noticed that when participants had their BP measured in the supine position, HW indicated that if the position was not posed as specified in the instruction, the measurement readings may be inaccurate (Figure 2).

### 3.2 ROC curve analysis

**FIGURE 3:**
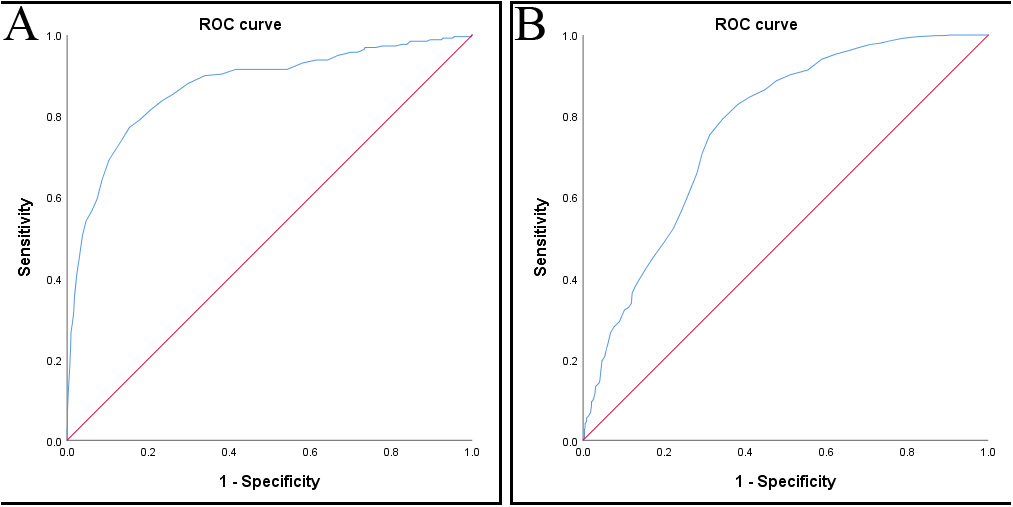
**(A)** ROC curve (set hypotension threshold < 80% PM baseline), **(B)** ROC curve (set hypotension threshold < 90% PM baseline). ROC, receiver operating characteristic.

When setting <80% of the baseline value of NIBP as the threshold for hypotension, the AUC was 0.87 (95% confidence interval: 0.85–0.90), *P* < 0.001 (two-tailed), Youden index = 0.62, cut-off point = 104 mmHg, (Figure 3A and Table 2a). The maximum Youden index is 0.62, corresponding to a HW value of 104 mmHg. Therefore, we set <104 mmHg as the threshold for HW hypotension.

**TABLE 2a:** Results of ROC (set hypotension threshold < 80% PM baseline).

AUC	*P*	Youden index	Cutoff point (mmHg)	95% CI	
				Lower	Upper
0.87	0.000	0.62	104	0.85	0.90

ROC, receiver operating characteristic; HW, HUAWEI WATCH D; NIBP, non-invasive blood pressure; AUV, area under the curve; CI, confidence interval.

When setting <90% of the baseline value of NIBP as the threshold for hypotension, the AUC was 0.77 (95% confidence interval: 0.74–0.80), *P* < 0.001 (two-tailed), Youden index = 0.45, cut-off point = 109 mmHg, (Figure 3B and Table 2b). The maximum Youden index is 0.45, corresponding to a HW value of 109 mmHg. Therefore, we set <109 mmHg as the threshold for HW hypotension.

**TABLE 2b:** Results of ROC (set hypotension threshold < 90% PM baseline).

AUC	*P*	Youden index	Cutoff point (mmHg)	95% CI	
				Lower	Upper
0.77	0.000	0.45	109	0.74	0.80

ROC, receiver operating characteristic; HW, HUAWEI WATCH D; NIBP, non-invasive blood pressure; AUV, area under the curve; CI, confidence interval.

### 3.3 Chi-squared analysis

The gold standard for hypotension was the SBP being <80% of the baseline value of the pressure monitor. For the HW, the results were as follows: sensitivity = 79.2%, specificity = 82.0%, false positive rate = 18.0%, false negative rate = 20.8%, and accuracy of 81.3%. Pearson χ^2^ = 350.4, *P* < 0.001, and Kappa = 0.53 (Table 3a). The gold standard for hypotension was the SBP being <90% of the baseline value of the pressure monitor. For the HW, the results were as follows: sensitivity = 56.5%, specificity = 88.9%, false positive rate = 11.1%, false negative rate = 43.5%, and accuracy of 73.0%. Pearson χ^2^ = 272.4, *P* < 0.001, and Kappa = 0.45 (Table 3b). Comparing the two hypotension thresholds, HW demonstrated markedly different diagnostic performance. At the 80% threshold, sensitivity was 79.2% with a specificity of 82.0%, yielding an AUC of 0.87 (95% CI: 0.85–0.90) and an overall accuracy of 81.3%. At the 90% threshold, sensitivity dropped to 56.5% and specificity rose to 88.9%. The AUC was 0.77 (95% CI: 0.74–0.80), and the accuracy was 73.0%. The superior Youden Index at an 80% threshold (0.62 vs. 0.45) indicates better overall diagnostic performance for detecting more severe hypotension. The Supplementary material can be found through the link at the end of the article.

**TABLE 3a:** Results of Chi-squared analysis (set hypotension threshold < 80% PM baseline).

HW	PM		Total count
	Hypotension	Normal SBP	
Hypotension	205	166	371
Normal SBP	54	754	808
Total count	259 (100.0)	920 (100.0)	1180 (100.0)

Pearson χ^2^ = 350.4, *P* < 0.001 (two-tails), Kappa = 0.53, *P* < 0.001 (two-tails). HW, HUAWEI WATCH D; SBP, systolic blood pressure; PM, pressure monitor. Sensitivity of 79.2%, specificity of 82.0%, false positive rate of 18.0%, false negative rate of 20.8%, and accuracy of 81.3%.

**TABLE 3b:** Results of Chi-squared analysis (set hypotension threshold < 90% PM baseline).

HW	PM		Total count
	Hypotension	Normal SBP	
Hypotension	327	67	394
Normal SBP	252	534	786
Total count	579 (100.0)	601 (100.0)	1180 (100.0)

Pearson χ^2^ = 272.4, *P* < 0.001 (two-tails), Kappa = 0.45, *P* < 0.001 (two-tails). HW, HUAWEI WATCH D; SBP, systolic blood pressure; PM, pressure monitor. Sensitivity of 56.5%, specificity of 88.9%, false positive rate of 11.1%, false negative rate of 43.5%, and accuracy of 73.0%.

## 4 Discussion

Compared with traditional mercury sphygmomanometers and NIBP and electronic BP monitor, the most significant advantage of HW are wearability and portability, which allows the measurement of BP anytime and anywhere. Although the instruction manual states that HW is suitable for BP screening rather than diagnosis, we compared the measurement of intraoperative hypotension between HW and NIBP to verify its accuracy in measuring hypotension.

The definition of intraoperative hypotension remains controversial in the academic community. According to the latest guidelines in the United Kingdom, the SBP of pregnant people undergoing cesarean section with spinal anesthesia should be maintained ≥90% of the baseline. However, a large-sample survey showed that less than 10% of anesthesiologists can meet this standard. Most anesthesiologists still default to using an SBP of <80% of the baseline as the criterion for intraoperative hypotension (2). Moreover, in recent years, almost all clinical anesthesia studies still use SBP <80% baseline or SBP < 90 or 100 mmHg as the threshold for hypotension (7, 12–22). So, we set <80% baseline as the hypotension threshold is in line with the mainstream. Our comparative analysis of the two thresholds reveals a necessary clinical trade-off. While recent UK guidelines recommend maintaining SBP ≥ 90% of baseline (11), HW’s sensitivity at this threshold was only 56.5%, indicating that 43.5% of hypotensive episodes would be missed. In contrast, HW performed substantially better at detecting the more severe 80% threshold (sensitivity 79.2%). This indicates that HW’s clinical utility may be confined to detecting significant hypotensive episodes, as opposed to the earlier, less severe BP reductions advocated by contemporary guidelines. This limitation further supports HW’s classification as a screening rather than a diagnostic tool for the management of intraoperative hypotension.

Recently, two studies have verified that the accuracy of HW in measuring SBP and DBP is comparable to that of mercury sphygmomanometers (1, 23). The incidence of spinal anesthesia-induced hypotension during elective cesarean section is reported to be as high as 70% – 90% (5, 6). Therefore, we adopted spinal anesthesia elective cesarean section for this study.

Compared with setting <90% as the hypotension threshold, setting <80% as the threshold and setting the HW hypotension threshold at 104 mmHg can demonstrate better sensitivity and accuracy in measuring supine hypotension. The aforementioned results indicate that although the monitoring effect of HW on supine hypotension is lower than that of NIBP, it still shows good monitoring accuracy. However, the 20.8% missed diagnosis rate may also lead to delayed treatment of maternal hypotension, resulting in rapid onset of supine hypotension syndrome symptoms such as nausea, vomiting, dizziness, cold sweats, difficulty breathing, tachycardia, tinnitus (24). Therefore, anesthesiologists should not only observe the BP value on HW, but also comprehensively judge the changes in BP based on the signs of maternal hypotension. A false negative rate of 20.8% means that approximately one in five hypotensive episodes would go undetected if HW were used as the sole monitoring device. Given the critical importance of prompt hypotension management during caesarian section to prevent maternal complications and fetal compromise, HW should be classified as a screening tool rather than a diagnostic monitor for intraoperative use. Therefore, HW cannot replace standard NIBP monitoring during surgery but may serve as an adjunct device in settings where continuous monitoring is unavailable or for postoperative surveillance.

We speculated that the possible reasons for the difference were as follows:

According to the HW manual and recent reports, the wearer should sit still, place the arm wearing HW at heart level and remain motionless during measurement. However, parturients who undergo elective cesarean section lay supine on the operating table, with both upper limbs outstretched at the same level as the heart. The HW displayed the prompt that “If you did not measure in the posture shown in the diagram, the measurement result may be inaccurate.” Thus, the difference in measurement position may be an important reason for inconsistency with the previous reports (1, 4).

Compared with standard sitting position, the SBP measured in supine position increased by 9.5 mmHg, in the supine position, compared with the position where the arm is outstretched and raised 5 cm to the same level as the right atrium, the SBP measured when the arm is directly placed on the examination bed increases by 4.6 mmHg (25). Applying these published estimates to our study, HW measurements in the supine position with arms on armrests may have been systematically elevated by approximately 14 mmHg (9.5 mmHg from supine posture + 4.6 mmHg from arm position). This systematic bias could partially explain the 18.0% false-positive rate, in which HW readings appeared artificially elevated relative to true hypotension. Future studies should use adjustable arm supports to maintain the wrist at heart level and directly measure the magnitude of this positional bias in pregnant women undergoing caesarian section. There is a need to refine the algorithm specifically for supine positioning and pregnancy-related hemodynamic changes before we can recommend HW for routine obstetric use.

According to general physiological principles, arterial BP is higher in the distal parts of the limbs than in the proximal arteries. HW measures BP at the wrist, whereas NIBP measures it at the upper arm. This difference in measurement sites therefore leads to discrepancies in the results obtained. The measurement time of HW (35–40 s) is longer than that of the NIBP (25–30 s). Consequently, these two BP monitoring modalities inherently exhibit variability when capturing hemodynamic parameters during distinct temporal windows.

There are some limitations in this study. The HW is designed and validated for seated measurements with the arm at heart level (Figure 2B), yet measurements were taken supine with arms outstretched. This is the fundamental limitation for low sensitivity of HW in measuring low BP. The sensor and BP algorithm of the watch may be designed for the natural position of the arm in front of the chest when sitting.

Several specific improvements are needed for pregnant women, who often have limited mobility and prefer supine BP monitoring, before HW can be recommended for clinical use in obstetrics. First, the oscillometric algorithm should be recalibrated for supine positioning, incorporating correction factors for hydrostatic pressure differences when the wrist is level with the body rather than elevated. Second, pregnancy-specific validation is needed given the physiological changes in arterial compliance, cardiac output, and sympathetic tone during gestation. Third, future validation studies should follow AAMI/ESH/ISO guidelines by performing same-arm sequential measurements (wrist vs. upper arm) rather than bilateral measurements. Finally, the device could incorporate position-detection sensors to alert users when measurements are taken outside validated conditions and apply appropriate correction algorithms.

According to American National Standards Institute/AAMI/ISO 81060-2:2018 guidelines, sequential BP measurements should be performed on the same arm (1, 26, 27). We will explore the comparison and correlation of hypotension readings between ipsilateral wrist HW and upper arm cuff in the future study.

Limited generalizability: the volunteers included in this study were healthy full-term parturients, and the accuracy of HW in measuring supine hypotension after spinal anesthesia in high-risk parturients with gestational hypertension, pregnancy complicated with heart disease, pregnancy complicated with thyroid dysfunction or pregnancy complicated with nephrotic syndrome, etc., was not evaluated.

## 5 Conclusion

The HUAWEI WATCH D demonstrates moderate diagnostic accuracy for detecting severe hypotension (SBP < 80% baseline) during caesarian section under spinal anesthesia, with a sensitivity of 79.2%, a specificity of 82.0%, and an AUC of 0.87. However, performance deteriorates substantially at the milder 90% threshold (sensitivity 56.5%), limiting its clinical utility for guideline-concordant hypotension management. The 20.8% false negative rate at an 80% threshold and positioning-related measurement bias when used supine (outside validated conditions) preclude HW’s use as a standalone intraoperative diagnostic monitor. HW should be classified as a screening tool rather than a replacement for standard NIBP monitoring during caesarian section. Potential applications include postoperative surveillance, home BP monitoring in high-risk obstetric patients, or adjunctive monitoring in resource-limited settings where continuous NIBP is unavailable.

## Data Availability

The original contributions presented in this study are included in this article/Supplementary material, further inquiries can be directed to the corresponding authors.
